# Effects of cold temperatures on abrasion resistance of concrete with different mixture compositions

**DOI:** 10.1038/s41598-026-56239-5

**Published:** 2026-06-10

**Authors:** Mohamed K. Ismail, Assem A. A. Hassan, Osama E. Shehata

**Affiliations:** 1 Department of Structural Engineering, Faculty of Engineering, Cairo University, Giza, Egypt; 2 Department of Civil Engineering, Faculty of Engineering and Applied Science, Memorial University of Newfoundland, St. John’s, NL Canada

**Keywords:** Abrasion resistance, Cold temperatures, Infrastructure, Aggregate characteristics, Binder content, Supplementary cementitious materials, Engineering, Materials science

## Abstract

This study investigates the abrasion resistance of concrete used in cold-region infrastructure, with a focus on the effects of temperature and mixture composition. A number of self-consolidating concrete (SCC) and normal concrete (NC) mixtures was evaluated under four temperature conditions (+ 20 °C, 0 °C, − 10 °C, and − 20 °C). The mixtures incorporated varying supplementary cementing materials (SCMs), namely metakaolin (MK), silica fume (SF), slag (SL), fly ash (FA), aggregate sizes, coarse-to-fine (C/F) aggregate ratios, and binder contents. Abrasion resistance was assessed using rotating-cutter and sandblasting test methods to provide a comprehensive evaluation of surface durability. The results indicate that mixtures containing MK and SF achieved the highest compressive strength and abrasion resistance, while FA showed the poorest performance; SL provided moderate strength gains but limited abrasion improvement. Due to denser aggregate packing from mechanical vibration, NC consistently outperformed SCC at all temperatures. Regarding mix design trade-offs, increasing the C/F aggregate ratio or maximum aggregate size reduced compressive strength but enhanced abrasion resistance. Lowering the binder content from 500 to 250 kg/m³ caused the largest overall performance losses. Decreasing temperature consistently enhanced both compressive strength and abrasion resistance across all mixtures. This enhancement was more pronounced in mixtures with low binder content or FA, whereas mixtures with refined MK and SF exhibited smaller relative gains but maintained superior absolute performance. The findings emphasize the interactive effects of temperature and mix design variables on abrasion performance, offering practical insight for developing concrete mixtures suited to cold-region infrastructures.

## Introduction

 The abrasion resistance of concrete is a key performance property that governs the durability and service life of infrastructure, particularly bridge decks, highways, airport runways, sidewalks, and road surfaces^1,2^. This property becomes particularly critical in cold regions, where concrete surfaces are subjected to intensified abrasive actions that accelerate deterioration. These actions arise from multiple mechanical sources: continuous friction from vehicle tires—especially heavy trucks and vehicles equipped with studded tires^3^—generates significant surface wear, while snowplow blades scraping against pavement during winter maintenance contribute to severe abrasion, particularly when improperly adjusted or when protective snow layers are absent. Additional abrasive forces stem from the grinding effect of sand and de-icing materials spread on roadways, which act as loose abrasive particles under traffic loading, as well as debris such as gravel, crushed stone, and dirt carried by vehicles. Beyond roadway surfaces, bridge piers and substructure elements in rivers and waterways are exposed to abrasive forces from moving sand, gravel, rocks, and ice flows, which erode concrete surfaces over time and lead to pitting, scaling, and material loss^4–6^. Collectively, these mechanical actions induce progressive material loss, surface roughening, and the formation of ruts or grooves^6–8^. Over time, such deterioration diminishes the long-term durability and service life of concrete infrastructure. Therefore, understanding the range of abrasion forces acting on concrete surfaces, as well as how concrete composition influences its response, is essential for designing durable mixtures capable of withstanding harsh service conditions in cold climates.

A review of the literature reveals that the mechanical properties of concrete at low temperatures have received limited research attention. Overall, existing studies indicate that lower temperatures tend to enhance mechanical performance. Lee et al.^9^ investigated compressive strength, splitting tensile strength, modulus of elasticity, and bond strength at temperatures ranging from 20 °C to − 70 °C, reporting increases in all properties as temperature decreased. However, they also found that cyclic temperature variations from freeze-thaw conditions reduced these properties, emphasizing the need to account for temperature effects in cold-region infrastructure design. Similarly, Krstulovic-Opara^10^ examined concrete behavior at cryogenic temperatures down to − 160 °C and observed increases in compressive and tensile strengths, elastic modulus, and Poisson’s ratio, along with reduced creep. These improvements were strongly influenced by moisture content, with dense aggregates enhancing strength gains while lightweight aggregates limited them. Stress-strain behavior became more linear and brittle at extreme cryogenic temperatures, though ultimate strain remained higher than at room temperature. Xie and colleagues^11^ investigated the axial compression performance of plain and reinforced concrete at temperatures from 20 °C to − 160 °C, reporting that as temperature decreased, concrete strength and elastic modulus increased while peak strain decreased, indicating increased brittleness. Extending these findings, MacLean and Lloyd^12^ demonstrated that compressive strength and elastic modulus increased linearly as temperature decreased from 20 °C to − 70 °C, with maximum compressive strength gains of approximately 40% at the lowest temperature, while peak strain showed statistically insignificant changes—further confirming the trend toward greater brittleness. The mechanisms underlying these low-temperature enhancements have been explored through microstructural analysis. Xie and Yan^13^ investigated the compressive strength of normal-weight concrete at temperatures from 20 °C to − 165 °C, examining the effects of water-to-cement ratio and water content. Their results showed that compressive strength increased almost linearly with decreasing temperature, reaching a maximum increase of 97% at the lowest temperature. Scanning electron microscope analysis revealed that low temperatures induced ice formation within the pore structure, which filled voids and contributed to the observed strength gain. Notably, water content significantly influenced the magnitude of strength improvement, with higher water content leading to greater increases at temperatures below − 40 °C. This finding was also highlighted by Zaki^6^, who investigated the abrasion resistance of fiber reinforced concrete at temperatures ranging from 20 °C to − 20 °C. Their results showed that strength and abrasion resistance improved with decreasing temperature although the effect was more pronounced in saturated specimens than in unsaturated specimens and was strongly influenced by aggregate gradation and binder content. Furthermore, the type length and volume of steel fibers significantly affected performance under cold temperatures with higher fiber contents and hooked end fibers providing the greatest enhancement in abrasion resistance and mechanical properties. Zhang et al.^14^ also investigated mechanical properties under varying water saturation and low-temperature conditions relevant to liquefied natural gas storage, reporting that while compressive and tensile strengths decreased with increasing water content at 20 °C due to water softening, they increased with water content at subzero temperatures due to ice formation within the pore structure; significant strength gains and increased brittleness occurred between − 30 °C and − 90 °C as water in nanopores froze. The dynamic behavior of concrete at low temperatures has also been examined. Su et al.^15^ investigated dynamic compressive behavior at temperatures from 20 °C to − 30 °C, finding that dynamic compressive strength increased by an average of 18% at − 30 °C compared to 20 °C, with an exponential strain rate strengthening effect. Damage under impact loading occurred primarily in the interfacial transition zone, mortar, and ice particles, with the interfacial transition zone being the most severely affected. Ismail and Hassan^16^ also investigated the behavior of concrete under drop-weight impact loading at temperatures ranging from 20 °C to − 20 °C, finding that impact resistance improved as temperature decreased, particularly below 0 °C. The same authors also evaluated the abrasion and impact resistance of their mixtures before and after freeze-thaw cycles in the presence of deicing salts^7^. They reported that freeze-thaw exposure reduced both impact and abrasion resistance, particularly in mixtures with low binder content and high coarse aggregate content. Supplementary cementing materials (SCMs) significantly influenced performance, with silica fume and metakaolin showing the highest resistance while fly ash showed the lowest. Kim et al.^17^ examined the effect of cryogenic temperature (− 170 °C) on normal concrete and ultra-high-performance fiber-reinforced concrete (UHPFRC) for liquefied natural gas storage applications, noting that while both materials exhibited increased compressive strength at low temperatures, normal concrete showed brittle behavior and reduced tensile performance after exposure, whereas UHPFRC demonstrated significant improvements in tensile strength, stiffness, and energy absorption. Lee and Choi^18^ similarly reported increases in strength and stiffness as temperature decreased from − 60 °C to 20 °C. Liu et al.^19^ examined the influence of low temperatures (− 50 °C to 30 °C) on mechanical behavior, showing that strength can increase by up to approximately 180% as pore water freezes into ice, restricting microcrack propagation, though this leads to higher brittleness and reduced ductility; a 42.7% increase in elastic modulus was also observed. Under dynamic loading, concrete exhibited nonlinear strength enhancement due to combined strain-rate hardening and viscoelastic energy dissipation, while temperatures below − 20 °C limited further strength gains and further reduced ductility. Chen et al.^20^ investigated the static mechanical properties, damage evolution, and sustainability of alkali-activated slag/fly ash recycled concrete at 20 °C, − 30 °C, − 60 °C, and − 90 °C, reporting that at − 90 °C, compressive, tensile, and flexural strengths increased by 65.35%, 51.52%, and 78.36%, respectively, while flexural toughness decreased by up to 19.98%. Similar trends of cold-temperature mechanical enhancement have been documented in additional studies^11,13,21–23^.

Notably, some studies have reported contradictory findings. Li et al.^24^ conducted uniaxial compressive tests on non-standard prismatic concrete specimens at temperatures of 20 °C, 0 °C, − 20 °C, − 30 °C, and − 40 °C, observing that as temperature dropped from 20 °C to 0 °C, compressive strength decreased while elastic modulus and peak strain increased; at temperatures below 0 °C, compressive strength rose, elastic modulus continued to grow, and peak strain decreased. These findings suggest that the effects of low temperature on strength may vary with specimen geometry, mix composition, and testing conditions. Furthermore, while static strength often improves at low temperatures, fatigue performance may deteriorate. Tian et al.^25^ conducted cyclic bending tests on UHPFRC prisms at 20 °C, − 10 °C, and − 20 °C, showing that low temperatures enhanced monotonic strength due to matrix stiffening but reduced flexural fatigue performance by increasing matrix brittleness and causing steel fiber fracture. As temperature decreased from 20 °C to − 10 °C and − 20 °C, flexural fatigue strength declined by 15.0% and 12.7%, respectively, while fatigue deformation accumulated more rapidly.

To advance understanding of concrete abrasion resistance under cold-temperature conditions, this study investigated the performance of both self-consolidating concrete (SCC) and normal concrete (NC) across a wide range of mixture parameters. Nine concrete mixtures were designed incorporating different supplementary cementing materials (fly ash (FA), slag (SL), silica fume (SF), and metakaolin (MK)), two coarse aggregate sizes (10 mm and 20 mm), varying coarse-to-fine (C/F) aggregate ratios (0.7 and 2.0), and two binder contents (250 kg/m³ and 500 kg/m³). The abrasion resistance was evaluated using both rotating-cutter and sandblasting methods, enabling a comprehensive assessment of surface durability under different wear mechanisms. Compressive strength was also tested. All tests were conducted at four temperatures: 20 °C, 0 °C, − 10 °C, and − 20 °C, with particular emphasis on subzero temperatures representative of northern terrestrial environments.

## Research significance

Concrete in cold-region infrastructure is exposed to subzero temperatures and severe abrasive actions, necessitating a thorough understanding of its performance under such conditions—particularly the combined influence of mixture composition and temperature—to enable the design of more durable and resilient systems. Unlike previous studies, the novelty of this work lies in:

- Evaluating concrete abrasion resistance and its dependence on the interaction between mixture composition and cold temperature conditions.
- Comparing two fundamentally different abrasion mechanisms, namely rotating cutter and sandblasting tests, to assess their relative aggressiveness and to better understand surface deterioration behavior in response to different abrasive actions.
- Applying statistical analysis to quantify the significance of the investigated parameters and temperature effects on strength and abrasion resistance.

The findings offer practical and scientifically grounded insights for optimizing concrete mix designs to better withstand mechanical wear in cold environments. Ultimately, this work contributes to improving the durability, safety, and service life of infrastructure in cold regions.

## Experimental program

### Materials

**Fig. 1:**
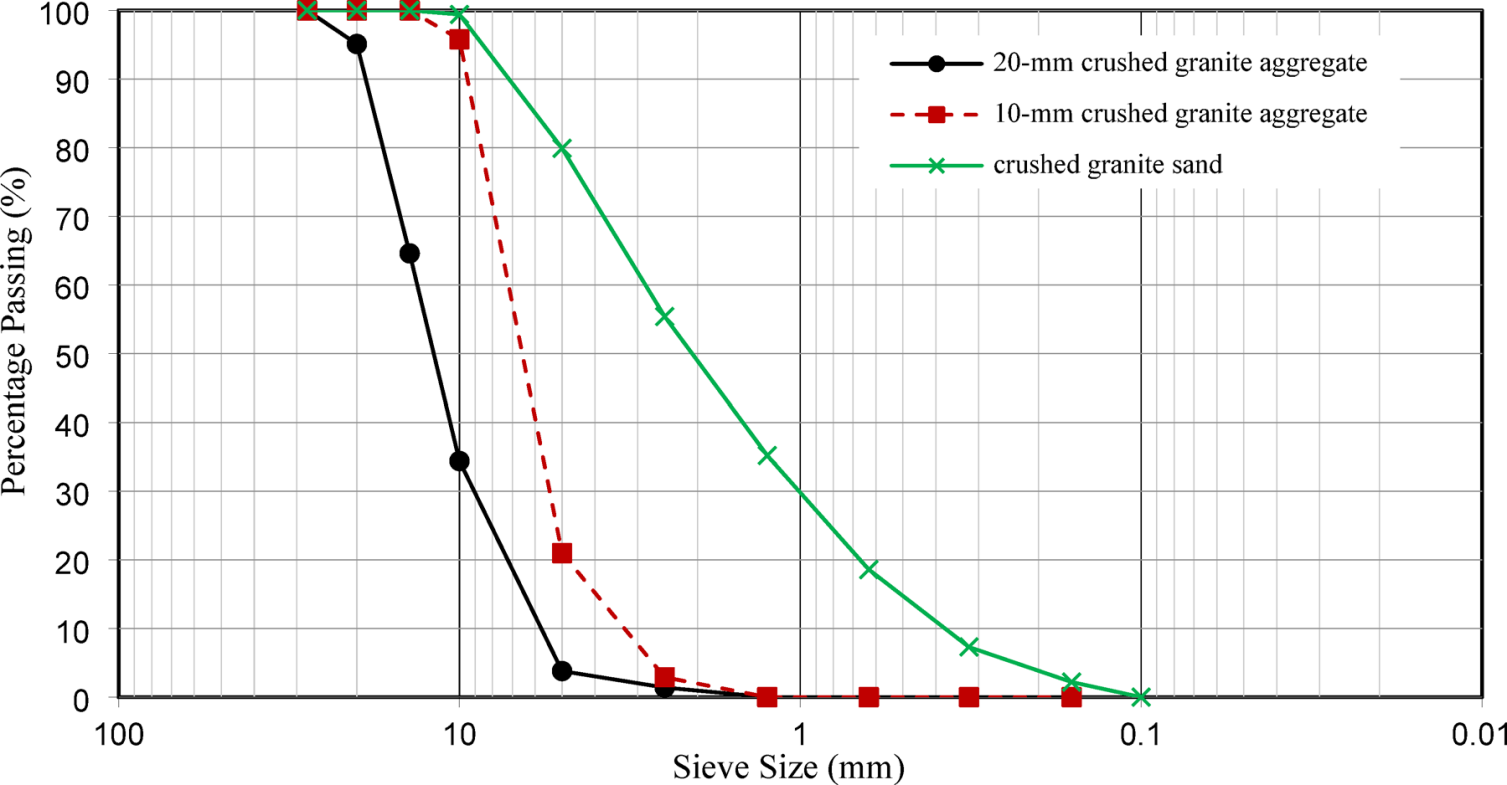
Gradation curves of aggregates.

Type I ordinary Portland cement, compliant with ASTM C150^26^, was used as the primary binding material in all concrete mixtures. Different SCMs—specifically FA, SL, SF, and MK—were used as partial replacements for cement, in accordance with ASTM C618^27^, ASTM C989^28^, ASTM C1240^29^, and ASTM C618^27^, respectively. Crushed granite was used for both coarse and fine aggregates, with their gradings presented in Fig. 1. A polycarboxylate-based high-range water-reducing admixture (HRWRA) conforming to ASTM C494^30^ was employed to achieve the desired workability.

### Developed mixtures

The experimental program comprised six SCC mixtures and three NC mixtures (see Table 1). The performance of SCC mixtures was optimized during the trial mixing stage to achieve compliance with the specifications of EFNARC (2005)^31^ for the SF2 class, which corresponds to a slump flow diameter ranging from 660 mm to 750 mm. The results indicated that a minimum w/b ratio of 0.4, a minimum binder content of 500 kg/m³, and a maximum C/F aggregate ratio of 0.7 were necessary to attain adequate flowability, passing ability, and stability. Based on these criteria, Mixture 1 was developed as an SCC with 500 kg/m³ cement, a w/b ratio of 0.4, a C/F aggregate ratio of 0.7, and a maximum aggregate size of 10 mm. Subsequently, Mixtures 2–5 were SCCs incorporating SCMs—30% FA, 30% SL, 8% SF, and 20% MK, respectively—as partial replacements for cement, based on optimal contents reported in previous studies^32–35^. These mixtures were designed to evaluate the influence of binder modification in SCC, while maintaining identical w/b ratio, total binder content, and aggregate size/content as Mixture 1 to ensure comparability of results. Additionally, Mixture 6 was prepared similarly to Mixture 1, with the only modification being an increase in maximum aggregate size to 20 mm, to evaluate the effect of aggregate size without altering other mixture constituents. The fresh properties of the developed mixtures were summarized in Table 2.

The flowability, passing ability, and stability of SCC were adversely affected by either a reduction in binder content or an increase in the C/F aggregate ratio. To address these limitations, three NC mixtures were developed to investigate the effects of a higher C/F aggregate ratio and a lower binder content on abrasion resistance. All NC mixtures were proportioned to achieve a slump of 120 ± 30 mm, with proportions detailed in Table 2. In this regard, Mixture 7 served as the reference NC mixture and was developed similarly to Mixture 1, but without self-compactability requirements through the use of a lower HRWRA dosage, while compaction was achieved mechanically. This mixture was utilized to evaluate the performance of SCC with self-compactability and high flowability in comparison with NC having relatively limited workability and requiring mechanical compaction. Meanwhile, Mixtures 8 and 9 were developed similarly to Mixture 7, but with a higher C/F aggregate ratio of 2.0 and a reduced binder content of 250 kg/m³, respectively, to evaluate the effects of coarse aggregate volume and binder content.

**Table 1:** Proportion details of tested mixtures.

Mix #	Mixture designation	Cement kg/m^3^	SCM Type	SCM kg/m^3^	C/F ratio	C.A. kg/m^3^	F.A. kg/m^3^	Water kg/m^3^	HRWRA (kg/m^3^)
1	SCC	500	-	-	0.7	686.5	980.8	200	2.4
2	SCC-30FA	350	FA	150	0.7	670.0	957.2	200	2.1
3	SCC-30SL	350	SL	150	0.7	682.1	974.5	200	2.3
4	SCC-8SF	460	SF	40	0.7	680.7	972.4	200	4.2
5	SCC-20MK	400	MK	100	0.7	678.7	969.6	200	5.4
6	SCC-S20	500	-–	-–	0.7	686.5	980.8	200	2.4
7	NC	500	-–	-–	0.7	686.5	980.8	200	0.9
8	NC-2 C/F	500	-–	-–	2	1111.5	555.8	200	0.9
9	NC-C250	250	-–	-–	0.7	771.5	1102.2	200	0.9

C. A. = coarse aggregate; F. A. = fine aggregate; HRWRA = high-range water-reducer admixture.

**Table 2:** Fresh properties of the developed SCC mixtures.

Mix #	Mixture designation	Slump flow	V-funnel (sec)	L-box
		T_50_ (sec)		
1	SCC-control	1.62	7.50	0.86
2	SCC-30FA	1.21	6.39	0.91
3	SCC-30SL	1.95	10.73	0.89
4	SCC-8SF	2.33	12.20	0.92
5	SCC-20MK	3.09	14.61	0.96
6	SCC-S20	1.42	8.96	0.81

### Testing program

All specimens were first moist cured for 28 days under controlled laboratory conditions. After curing, the specimens were stored at room temperature to allow moisture redistribution and stabilization, better approximating the moisture condition of in-service concrete prior to testing.

Subsequently, all specimens were conditioned at their target temperatures before testing. Specimens designated for testing at + 20 °C were stored and tested at room temperature, while those intended for 0 °C, − 10 °C, and − 20 °C were conditioned in three separate cooling rooms for 3 days to ensure thermal stabilization throughout the specimen volume. Thermal equilibrium was ensured by maintaining a constant room temperature and by selecting a conditioning duration based on specimen dimensions and preliminary trials. Immediately prior to testing, specimens were removed and tested without delay in the same laboratory, with an average testing duration of 2–3 min, in close proximity to the conditioning room to minimize temperature variation.

For all the following tests, three replicate specimens were tested for each mixture and temperature condition. The results are reported as mean values with corresponding standard deviations.

#### Compressive strength

The compressive strength was measured using three identical cylindrical specimens measuring 100 mm in diameter and 200 mm in height, in accordance with ASTM C39^36^.

#### Abrasion resistance tests

**Fig. 2:**
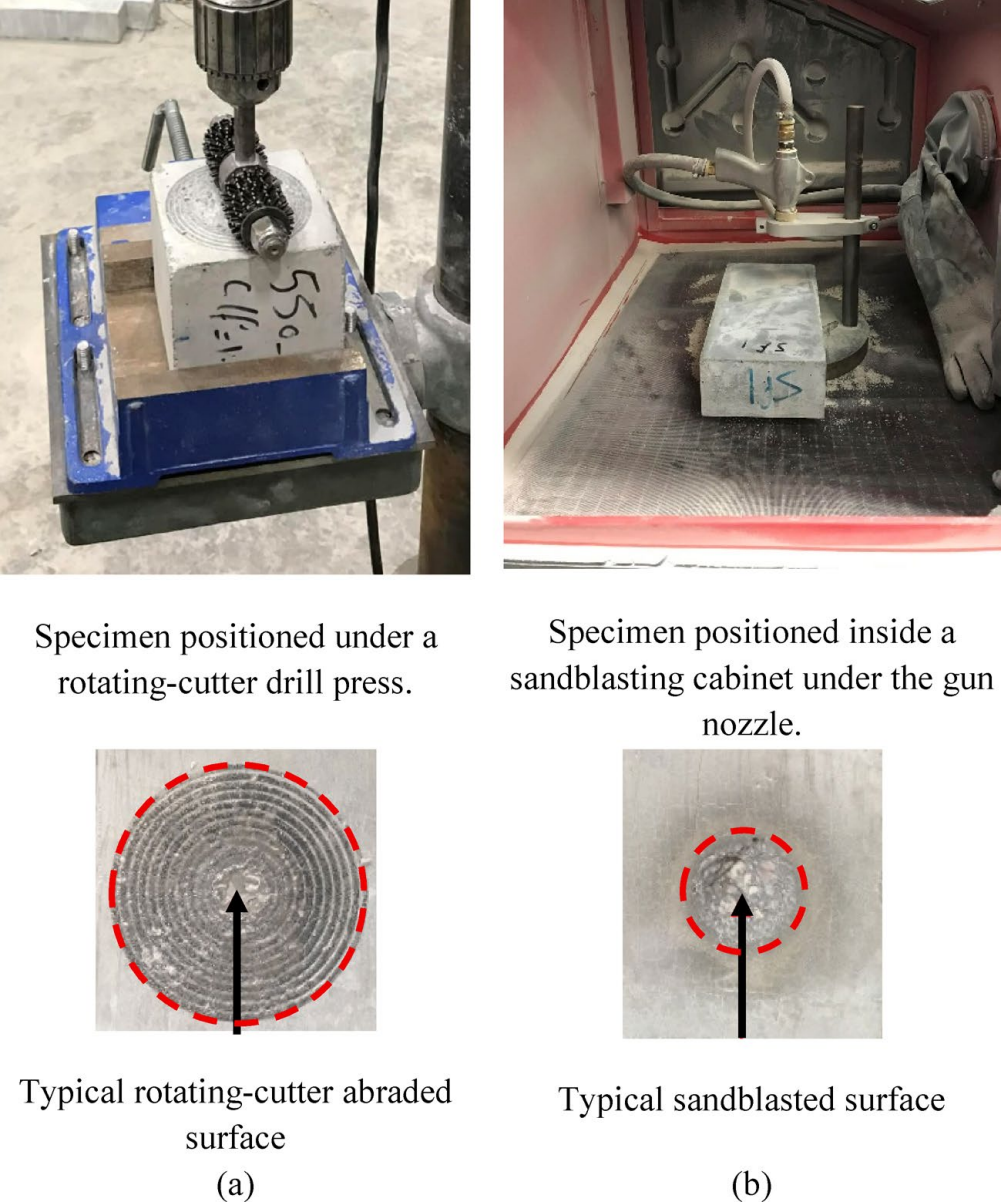
Abrasion tests (**a**) rotating cutter, (**b**) sandblasting.

The abrasion resistance of all developed mixtures was evaluated using two techniques (see Fig. 2), as follows:

(i) Rotating-cutter method: This test was conducted as per ASTM C944^37^, utilizing for quality control of bridge and highway concrete exposed to traffic. For each mixture, three cubic specimens measuring 100 mm were tested. Each specimen was mounted in a rotating-cutter drill press and subjected to a two-minute abrasion cycle at 200 rpm under a constant 98 N load. After each cycle, the specimen surface was cleaned of debris, and its mass was measured to the nearest 0.1 g. The mass loss for each specimen was determined, and then the equivalent average depth of wear was calculated.
(ii) Sandblasting method: This test was carried out in accordance with ASTM C418^38^, simulating the effects of waterborne abrasives and moving traffic on concrete surfaces. In this test, the specimen was positioned inside the sandblast cabinet normal to the nozzle axis, at a distance of 75 ± 2.5 mm from the nozzle tip. The concrete surface was blasted with air-driven silica sand for one minute, repeated on at least eight distinct locations. Afterward, the abrasion cavities were measured to determine the abraded volume. The abrasion coefficient (*A*_*c*_) (i.e., equivalent average depth) was calculated using Eq. (1) to the nearest 0.01 cm³/cm²:

1 $${A_c}={\text{ V }}/{\text{ A}}$$

where *A*_*c*_ is the abrasion coefficient (cm³/cm²), V is the abraded volume (cm³), and A is the abraded surface area (cm²).

## Results and discussion

### Compressive strength

#### Effect of SCMs

**Fig. 3:**
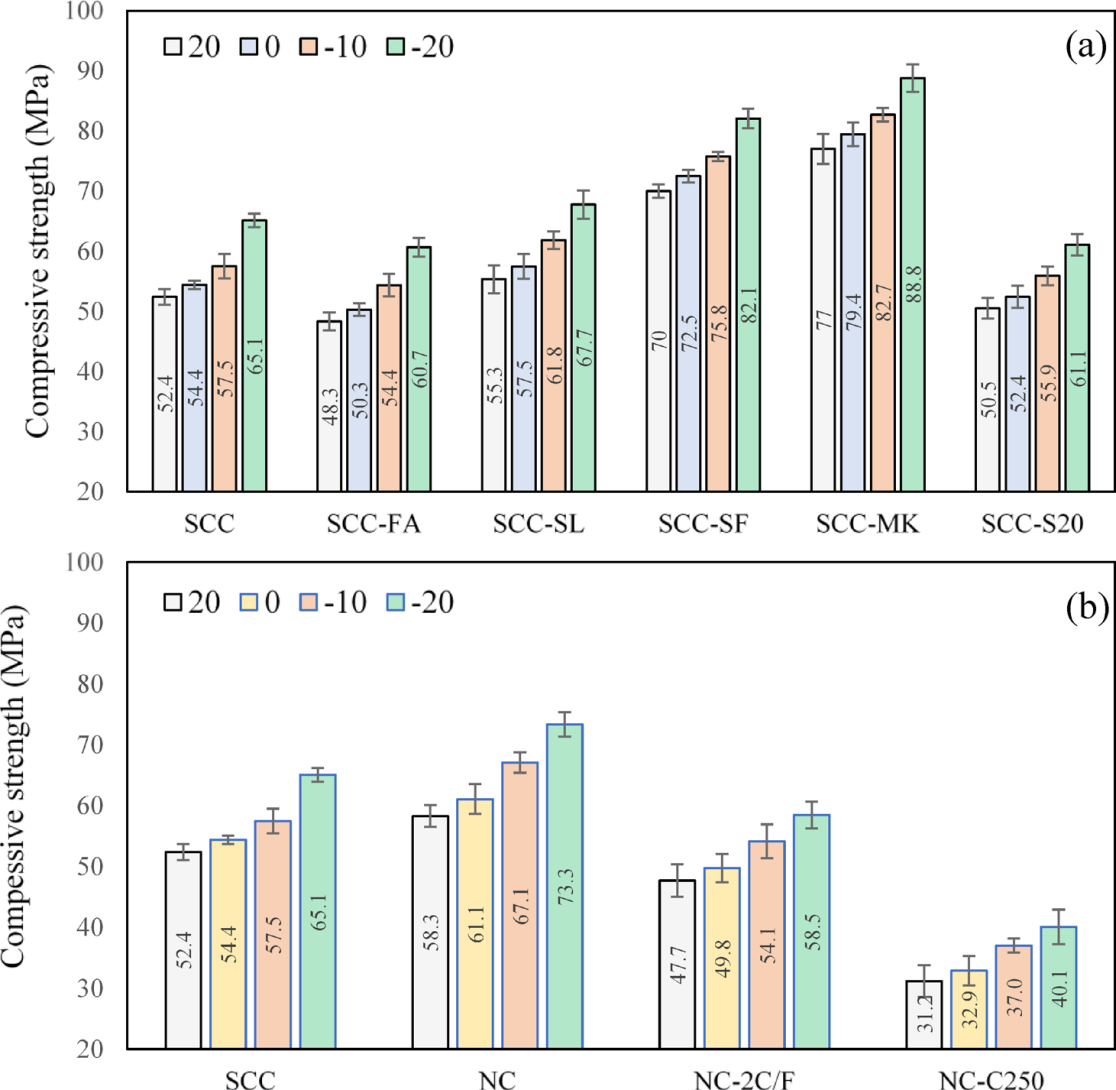
Compressive strength results (**a**) effect of SCMs and aggregate size, (**b**) effect of concrete type, C/F aggregate ratio, and binder content.

As shown in Fig. 3a, at room temperature (+ 20 °C), the control SCC achieved a compressive strength of 52.4 MPa. The incorporation of 30% FA in SCC-30FA mixture resulted in a strength of 48.3 MPa, representing a 7.8% reduction, which is attributed to the slow pozzolanic reactivity of FA compared to cement, which limits its contribution to concrete strength and microstructure at early ages, with most improvements becoming evident at later ages^39–42^. In contrast, SCC-30SL mixture with 30% SL achieved 55.3 MPa, a 5.5% increase over the control mixture, due to slag’s reactive properties, which form extra calcium silicate hydrate (C-S-H) gel, densifying the microstructure and enhancing strength development^42,43^. Vejmelkova et al.^44^ reported that XRD analysis revealed a significant decrease in calcium hydroxide content in concrete containing SL over time, with the formation of C-S-H filling pores and making the concrete’s internal structure denser. More notably, SCC-8SF mixture incorporating 8% SF reached 70.0 MPa, a substantial 33.6% increase, while SCC-20MK with 20% MK exhibited the highest strength at 77.0 MPa, representing a 47.0% increase compared to the control SCC mixture. These improvements are attributed to the high pozzolanic activity of SF and MK, which refine the pore structure, strengthen the interfacial transition zone (ITZ), and promote dense C-S-H formation^45,46^.

As the temperature decreased, all mixtures showed progressive increases in compressive strength. At 0 °C, SCC, SCC-30FA, SCC-30SL, SCC-8SF, and SCC-20MK reached 54.4 MPa, 50.3 MPa, 57.5 MPa, 72.5 MPa, and 79.4 MPa, respectively, representing increases of 3.8%, 4.1%, 3.9%, 3.5%, and 3.2% compared to their strengths at 20 °C. At -10 °C, further increases were observed: the control SCC rose to 57.5 MPa (9.7% increase), SCC-30FA achieved 54.4 MPa (12.5% increase), SCC-30SL reached 61.8 MPa (11.8% increase), SCC-8SF attained 75.8 MPa (8.2% increase), and SCC-20MK increased to 82.7 MPa (7.4% increase). At the lowest testing temperature of -20 °C, all mixtures exhibited substantial increases in compressive strength. The control SCC reached 65.1 MPa, a 24.2% increase; SCC-30FA recorded 60.7 MPa, up 25.7%; SCC-30SL attained 67.7 MPa, a 22.4% increase; SCC-8SF achieved 82.1 MPa, up 17.3%; and SCC-20MK reached 88.8 MPa, corresponding to a 15.3% gain. These increases, as reported in the literature^9,47,48^, may be attributed to the formation of ice within the cement paste, which can partially fill pores and air voids while mitigating defects such as microcracks and weaknesses in the interfacial transition zone. These interpretations were supported by scanning electron microscope analyses reported in^13^, which indicated that low temperatures promoted ice formation within the pore structure, thereby filling voids and contributing to the observed strength gain. In addition, cold-induced shrinkage has been hypothesized in the literature to reduce atomic spacing, which may enhance interatomic bonds and improve the internal cohesion of the concrete^49,50^. The results also showed that mixtures containing SF or MK exhibited smaller increases in compressive strength compared to mixtures incorporating FA or SL. This behavior may be related to the higher pozzolanic reactivity and finer particle size of SF and MK relative to cement, FA, and SL, which generally contribute to the development of a denser and more refined microstructure. As a result, the reduced pore volume and smaller interparticle spacing may limit the degree of strength enhancement associated with ice formation at low temperatures. Nevertheless, the ranking of SCM performance remained consistent across all testing temperatures, with MK and SF consistently outperforming SL and FA due to their superior pozzolanic reactivity and microstructural refinement.

#### Effect of coarse aggregate size

Figure 3b illustrates the interaction between coarse aggregate size and temperature. At 20 °C, SCC-S20, with a larger maximum coarse aggregate size of 20 mm, exhibited a compressive strength of 50.5 MPa, slightly lower than the 52.4 MPa of the control SCC with 10 mm aggregates, representing a 3.6% reduction likely due to the larger aggregate size and the associated weaker ITZ. Decreasing temperature led to strength gains regardless of aggregate size. At 0 °C, − 10 °C, and − 20 °C, the strength of SCC-S20 (20 mm aggregate) increased by 3.8%, 10.7%, and 20.9%, respectively, compared to control SCC (10 mm aggregate), which showed gains of 3.8%, 9.7%, and 24.2%. The strength gap between the two mixtures remained relatively consistent across all temperatures, with the control SCC exhibiting 3.2–4.0 MPa higher strength throughout.

#### Effect of mixture type (SCC vs. NC)

The temperature-dependent interaction with concrete type (NC vs. SCC) is demonstrated by Fig. 3b. At 20 °C, the control NC mixture achieved a compressive strength of 58.3 MPa, exceeding the control SCC by 11.3% (5.9 MPa), as shown in Fig. 3b. This higher strength may be attributed to the NC being mechanically vibrated, which leads to better compaction and reduced voids, whereas the SCC relies on self-compaction and may retain more entrapped air. As the temperature decreased, both NC and SCC mixtures exhibited progressive increases in compressive strength. At 0 °C, NC increased by 4.8% from its 20 °C value, while SCC gained 3.8%. At − 10 °C, NC rose by 15.1% compared to 9.7% for SCC. At − 20 °C, NC recorded a 25.7% increase versus 24.2% for SCC. The strength advantage of NC over SCC grew slightly at lower temperatures, from 5.9 MPa at 20 °C to 8.2 MPa at − 20 °C. This may be because, in well-compacted concrete like NC, closely packed particles make cold-induced shrinkage more effective at enhancing interatomic bonds and internal cohesion. In addition to compaction effects, SCC—unlike NC—is characterized by high flowability (achieved by incorporating high dosages of HRWRA), which can cause cement paste and fine particles to migrate toward the surface, forming a paste-rich mortar layer with a lower concentration of coarse aggregate. Since coarse aggregate is the primary contributor to abrasion resistance and surface hardness, this can make the surface more vulnerable to wear. Additionally, the higher HRWRA in SCC, in some cases, can delay early hydration and setting at the surface, resulting in a weaker near-surface layer and a comparatively softer wearing surface than that of normal concrete.

#### Effect of C/F aggregate ratio

The effect of C/F aggregate ratio across all temperatures is shown in Fig. 3b. At a temperature of 20 °C, NC-2 C/F, which developed with a higher C/F aggregate ratio of 2.0, exhibited a compressive strength of 47.7 MPa, representing a significant 18.2% reduction compared to that of the control NC (which developed with a C/F aggregate ratio of 0.7), which had a compressive strength of 58.3 MPa. As temperature decreased, NC-2 C/F exhibited consistent strength gains. When temperature dropped from 20 °C to 0 °C, − 10 °C, and − 20 °C, compressive strength increased by 4.3%, 13.5%, and 22.6%, respectively (Fig. 3b). By comparing the NC-2 C/F mixture with the control NC mixture, it can be observed that although both mixtures benefited from reduced temperatures, the NC-2 C/F mixture exhibited a less pronounced rate of strength development compared to the control NC. The strength gap between NC-2 C/F and the control NC widened from 10.6 MPa at 20 °C to 14.8 MPa at − 20 °C. This indicates that mixtures with higher mortar content may be more sensitive to cold temperatures. This behavior can be explained by the fact that the mechanisms induced by low-temperature exposure (i.e., ice formation and increased interatomic bonds), as discussed previously, primarily occur within the mortar phase. Therefore, a larger mortar volume results in a more pronounced influence of cold temperature on the overall behavior of the mixture.

#### Effect of binder content

As shown in Fig. 3b, at temperature of 20 °C, NC-C250, with a lower binder content of 250 kg/m³, recorded a compressive strength of only 31.2 MPa, representing a substantial 46.5% reduction compared to the control NC mixture with a binder content of 500 kg/m³. This reduction is primarily attributed to the limited formation of hydration products due to the lower cementitious materials, which are essential for strength development. Additionally, the reduced binder leads to poorer particle packing, inadequate coating of aggregate particles, and weaker interparticle bonding. These factors collectively result in a less cohesive matrix, ultimately causing a decline in the overall strength of the concrete composite.

The strength of the NC-C250 mixture increased as the temperature decreased, showing gains of 5.5%, 18.6%, and 28.5% at 0 °C, -10 °C, and − 20 °C, respectively. Although NC-C250 exhibited one of the highest proportional increases, its absolute strength remained the lowest among all mixtures at all temperatures, indicating that the strengthening effect of subzero temperatures could not compensate for the low binder content. Comparing NC-C250 with the control NC reveals that the strength difference between them was 27.1 MPa at 20 °C, rising to 33.2 MPa at -20 °C. This confirms the previously discussed observation that the positive effect of cold temperatures on concrete strength becomes more pronounced as mortar volume increases.

### Abrasion resistance

#### Effect of SCMs

**Fig. 4:**
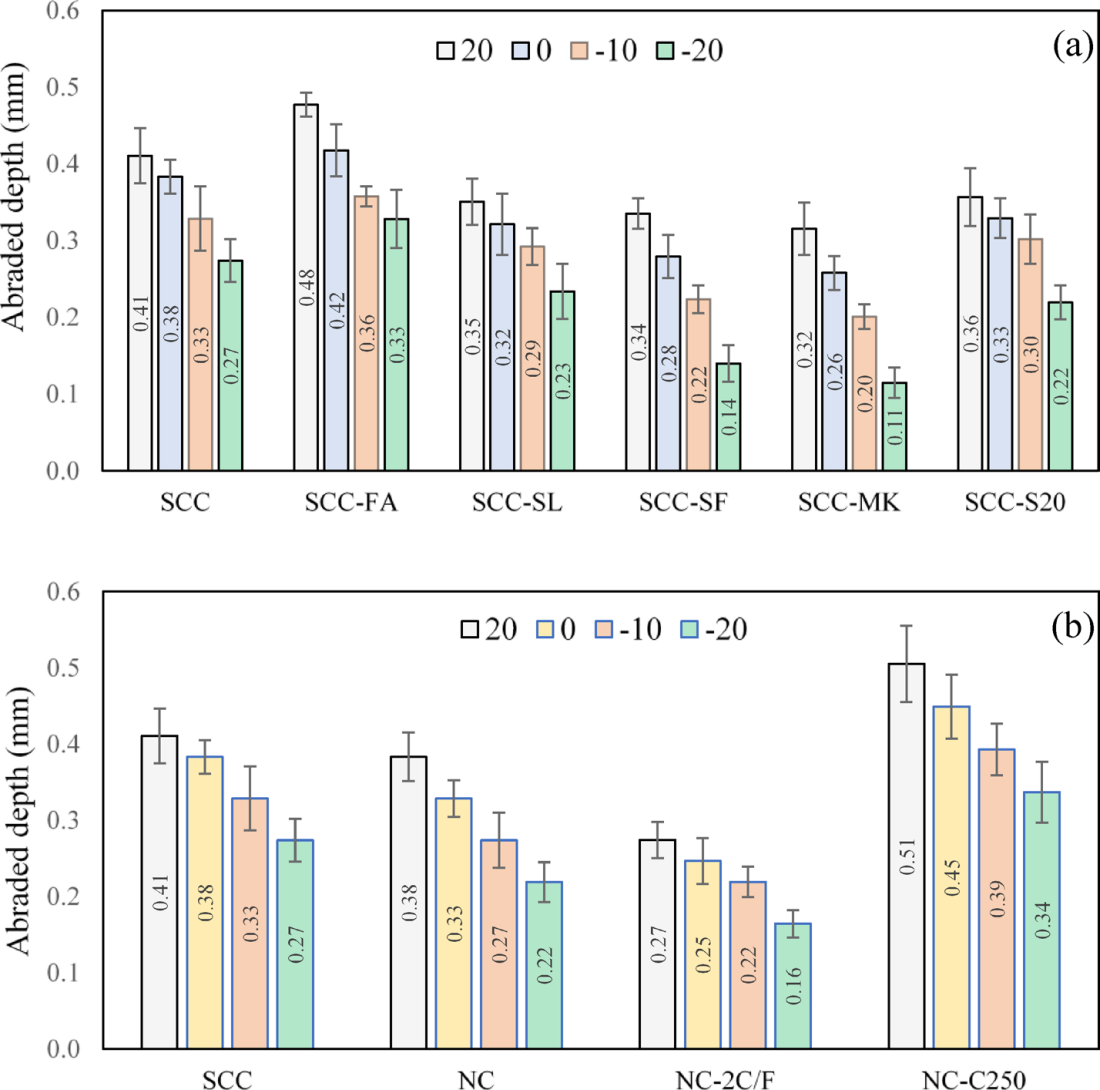
Rotating cutter test results (**a**) effect of SCMs and aggregate size, (**b**) effect of concrete type, C/F aggregate ratio, and binder content.

**Fig. 5:**
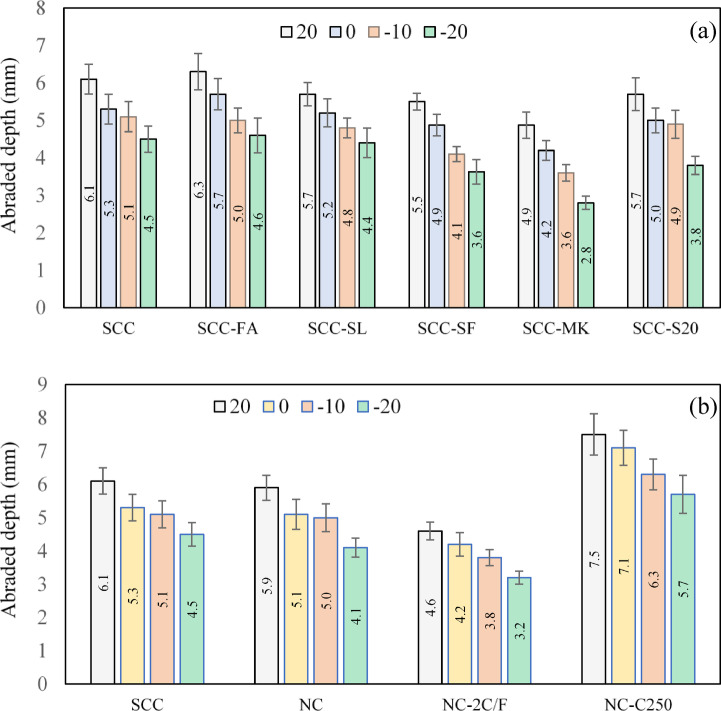
Sandblasting test results (**a**) effect of SCMs and aggregate size, (**b**) effect of concrete type, C/F aggregate ratio, and binder content.

Figures 4a and 5a show the average abraded depth of all SCMs mixtures recorded from both rotating-cutter and sandblasting tests. At a temperature of 20 °C, the control SCC mixture exhibited an abraded depth of 0.41 mm in the rotating cutter test and 6.1 mm in the sandblasting test. It is worth noting that the difference in equivalent average depth values (by an order of magnitude) obtained from the rotating-cutter and sandblasting methods is attributed to their distinct abrasion mechanisms. The rotating-cutter method induces gradual and distributed mechanical wear under controlled loading (as shown in Fig. 2a), representing field traffic conditions where pavement surfaces undergo progressive abrasion under vehicle loading, and thus results in lower depth values. In contrast, the sandblasting method imposes concentrated, high-energy localized abrasion via silica sand impact (as shown in Fig. 2b), representing severe field actions such as localized erosion from wind-blown or waterborne sand and debris, leading to pronounced mortar removal and higher depth values. Compared to the control SCC mixture, the incorporation of different SCMs affected the surface matrix hardness and abrasion resistance. Incorporating FA in SCC-FA increased abrasion depth in both test methods, with rotating cutter depth reaching 0.48 mm (a 17.1% increase) and sandblasting depth reaching 6.3 mm (a 3.3% increase), indicating that FA replacement weakened the matrix and made it more susceptible to abrasion due to its slower pozzolanic activity. In contrast, incorporating SG improved abrasion resistance. SCC-SG exhibited reduced abrasion depths of 0.35 mm (14.6% decrease) in the rotating cutter test and 5.7 mm (6.6% decrease) in the sandblasting test. Similarly, SCC-S20 showed moderate improvement, with values of 0.36 mm (12.2% decrease) and 5.7 mm (6.6% decrease), respectively. SCC-SF, incorporating SF, performed better than both SCC-FA and SCC-SG, recording rotating cutter depth of 0.34 mm (17.1% reduction) and sandblasting depth of 5.5 mm (9.8% reduction). SCC-MK exhibited the best performance among SCC mixtures, lowering the rotating cutter and sandblasting depths by 22.0% and 19.7% to 0.32 mm and 4.9 mm, respectively. The superior performance of SF and MK is attributed to their high pozzolanic reactivity and fine particle size, which densify the concrete matrix, fill voids, and refine the interfacial transition zone around aggregates. This results in a harder, more cohesive surface that resists particle dislodgement and wear.

Compared to the abraded depth measured in the rotating cutter test at 20 °C, all mixtures exhibited lower abraded depths at 0 °C, − 10 °C, and − 20 °C, confirming a consistent enhancement in abrasion resistance with decreasing temperature. This behavior may be attributed to the same mechanisms discussed previously for strength enhancement at low temperatures, including pore filling by ice formation and increased internal cohesion, which collectively improve surface hardness and reduce micro-scale material removal. In rotating cutter test, the control SCC mixture showed reduced abraded depths of 0.38, 0.33, and 0.27 mm, representing reductions of 6.7%, 20.0%, and 33.3%, respectively. Similarly, SCC-FA recorded abraded depths of 0.42, 0.36, and 0.33 mm, corresponding to reductions of 12.5%, 25.0%, and 31.3%, while SCC-SL showed values of 0.32, 0.29, and 0.23 mm, with reductions of 8.3%, 16.7%, and 33.3%. Unlike the compressive strength results, SCC-SF and SCC-MK mixtures exhibited the most pronounced improvements, with SCC-SF showing abraded depths of 0.28, 0.22, and 0.14 mm (reductions of 16.7%, 33.3%, and 58.3%), while SCC-MK achieved even greater reductions with values of 0.26, 0.20, and 0.11 mm (reductions of 18.2%, 36.4%, and 63.6%). These results indicated that the abrasion resistance, unlike compressive strength, may be more strongly influenced by the effect of low temperature on enhancing the internal cohesion and surface hardness of the material. In SCC-SF and SCC-MK, the denser and more refined microstructures produced by the high pozzolanic reactivity of SF and MK may contribute to stronger particle bonding and a more compact surface layer. Consequently, exposure to low temperatures may further increase the resistance of the surface to micro-scale material removal, resulting in improved abrasion resistance. In contrast, mixtures with comparatively less refined microstructures may exhibit a less pronounced enhancement in abrasion resistance under low-temperature conditions.

A similar trend was observed in the sandblasting test, although with higher abraded depth values due to its more aggressive wear mechanism. As temperature decreased from 20 °C to 0 °C, − 10 °C, and − 20 °C, all mixtures showed a progressive reduction in abraded depth, indicating enhanced resistance to particle impact erosion. The control SCC mixture decreased to 5.3, 5.1, and 4.5 mm, corresponding to reductions of 13.1%, 16.4%, and 26.2%, respectively. SCC-FA showed values of 5.7, 5.0, and 4.6 mm (reductions of 9.5%, 20.6%, and 27.0%). SCC-SG exhibited 5.2, 4.8, and 4.4 mm, with reductions of 8.8%, 15.8%, and 22.8%, reflecting moderate improvement. More pronounced reductions were observed in SCC-SF and SCC-MK. SCC-SF decreased to 4.9, 4.1, and 3.6 mm (11.4%, 25.5%, and 34.0% reductions), while SCC-MK achieved the lowest values of 4.2, 3.6, and 2.8 mm, corresponding to reductions of 13.8%, 26.1%, and 42.5%. This behavior confirms that mixtures with denser and more refined microstructures benefit more from temperature reduction.

Overall, although both test methods demonstrate improved abrasion resistance with decreasing temperature, the magnitude of improvement is consistently higher in the rotating cutter test than in the sandblasting test, suggesting that while ice formation significantly enhances resistance to surface grinding, its effectiveness is relatively reduced under high-energy particle impact. Nonetheless, mixtures with highly refined microstructures, particularly those containing SF and MK, maintain superior performance across both abrasion mechanisms, highlighting the combined influence of pore structure refinement and freezing-induced hardening.

#### Effect of coarse aggregate size

As shown in Figs. 4a and 5a, at 20 °C, SCC-S20—incorporating a larger maximum coarse aggregate size of 20 mm—exhibited a rotating cutter depth of 0.36 mm and a sandblasting depth of 5.7 mm, compared with 0.41 mm and 6.1 mm, respectively, for the control SCC developed with maximum coarse aggregate size of 10 mm. In the rotating cutter test, SCC-S20 showed a 13.1% improvement, whereas the sandblasting test indicated a more modest enhancement of 6.6%.

At cold temperatures, both mixtures exhibited improved abrasion resistance across both test methods. In the rotating cutter test, SCC-S20 exhibited abraded depths of 0.33 mm, 0.30 mm, and 0.22 mm at 0 °C, -10 °C, and − 20 °C, corresponding to increases of 7.7%, 15.4%, and 38.5% relative to 20 °C. The control SCC showed depths of 0.38 mm, 0.33 mm, and 0.27 mm, with increases of 6.7%, 20.0%, and 33.3%, respectively. In the sandblasting test, SCC-S20 recorded depths of 5.0 mm, 4.9 mm, and 3.8 mm at the same temperatures, corresponding to increases of 12.3%, 14.0%, and 33.3%, while the control SCC exhibited 5.3 mm, 5.1 mm, and 4.5 mm, with increases of 13.1%, 16.4%, and 26.2%, respectively.

These results indicate that SCC-S20 consistently outperforms the control SCC in the rotating cutter and sandblasting tests across all temperatures, highlighting the role of larger coarse aggregates as hard inclusions that enhance mechanical abrasion resistance. This effect is particularly pronounced at subzero temperatures, where the combination of rigid aggregates and an ice-reinforced matrix produced a highly durable surface.

#### Effect of mixture type (SCC vs. NC)

At 20 °C, the NC mixture exhibited rotating cutter and sandblasting depths of 0.38 mm and 5.9 mm (see Figs. 4b and 5b), respectively, compared with 0.41 mm and 6.1 mm for the control SCC. This corresponds to a 6.7% improvement in rotating cutter resistance and a 3.2% improvement in sandblasting resistance for NC over SCC. Although both mixtures were developed with the same composition, these differences can be attributed to the higher flowability of SCC, which tends to produce a surface layer richer in mortar, making it slightly more susceptible to abrasion than the NC mixture.

In cold temperatures, the NC mixture also outperformed the control SCC mixture in both test methods. For example, at -20 °C, NC recorded rotating cutter depth of 0.22 mm (42.9% reduction from 20 °C) and sandblasting depths of 4.1 mm (30.5% reduction), while the control SCC achieved rotating cutter depths of 0.27 mm (34.1% reduction) and sandblasting depths of 0.46 mm (25.8% reduction).

#### Effect of C/F aggregate ratio

Figures 4b and 5b also shows that as the C/F aggregate ratio increased abrasion resistance increased, unlike compressive strength results. At 20 °C, NC-2 C/F (C/F ratio of 2.0) exhibited rotating cutter and sandblasting depths of 0.27 mm and 0.46 mm, respectively, representing a 28.5% improvement in rotating cutter resistance and a 22.0% improvement in sandblasting resistance compared to NC with a C/F ratio of 0.7. These significant enhancements could be related to the increased coarse aggregate content, which provides a greater volume of hard, wear-resistant particles at the surface, thereby reducing the depth of abrasion.

As temperature decreased, NC-2 C/F showed progressively lower abrasion depths. Rotating cutter depths were 0.25 mm, 0.22 mm, and 0.16 mm at 0 °C, − 10 °C, and − 20 °C (reductions of 10.0%, 20.0%, and 40.0%), while sandblasting depths were 4.2 mm, 3.8 mm, and 3.2 mm (reductions of 8.7%, 17.4%, and 30.4%). These gains stem from ice formation stiffening the matrix and the high coarse aggregate content, which together enhance surface hardness under cold conditions.

#### Effect of binder content

Figures 4b and 5b shows the effect of binder content on abrasion resistance under all testing temperatures. At a temperature of 20 °C, NC-C250, with a reduced binder content of 250 kg/m³, recorded rotating cutter and sandblasting depths of 0.51 mm and 7.5 mm, respectively, representing increases of 34.2% and 27.1% compared with the reference NC (0.38 mm and 5.9 mm). This substantial deterioration in abrasion resistance is attributed to the insufficient cementitious paste volume, which increases surface porosity, weakens the bond between aggregates, and produces a less cohesive matrix that is more susceptible to both mechanical and erosive wear. The particularly severe increase in sandblasting depth highlights the vulnerability of low-binder mixtures to erosive abrasion, where the lack of a cohesive matrix allows rapid surface degradation.

As the testing temperature decreased, NC-C250 exhibited some improvement in abrasion resistance, but the abraded depths remained higher than those of the reference NC. At 0 °C, NC-C250 recorded rotating cutter and sandblasting depths of 0.45 mm (11.1% increase from 20 °C) and 7.1 mm (5.3% increase), compared with NC at 0.33 mm and 5.1 mm, respectively. At -10 °C, NC-C250 showed depths of 0.39 mm and 6.3 mm (22.2% and 16.0% increases), while NC exhibited 0.27 mm and 5.0 mm. At -20 °C, NC-C250 achieved 0.34 mm and 5.7 mm (33.3% and 24.0% increases), whereas NC reached 0.22 mm and 4.1 mm.

Despite the proportional improvements at lower temperatures due to the ice-reinforced matrix, NC-C250 consistently exhibited the highest abraded depths among all mixtures across both test methods. This highlights that insufficient binder content cannot be fully compensated by low-temperature effects, emphasizing that an adequate amount of cementitious material, in combination with coarse aggregates, is essential for producing concrete structures durable against both mechanical and erosive abrasive wear.

### Correlation of compressive strength, abrasion resistance, and temperature effects

**Fig. 6:**
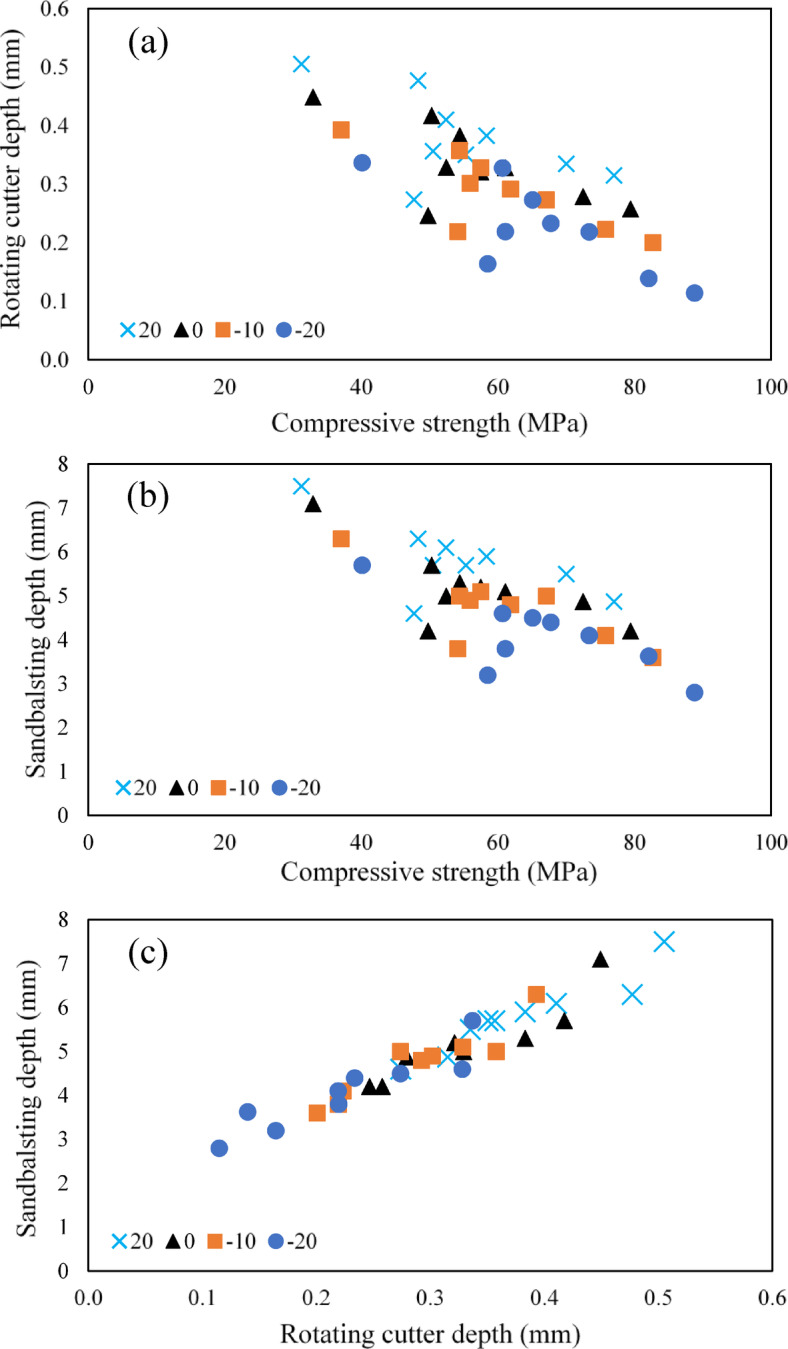
Correlation at different testing temperatures (20, 0, − 10, −20 °C): (**a**) compressive strength vs. rotating cutter depth, (**b**) compressive strength vs. sandblasting depth, and (**c**) rotating cutter depth vs. sandblasting depth.

Figure 6 illustrates the general correlation between compressive strength, rotating cutter depth, and sandblasting depth under different testing temperatures (20, 0, − 10, and − 20 °C). Figures 6a and b indicate a clear positive relationship between compressive strength and abrasion resistance, where an increase in compressive strength corresponds to a reduction in abraded depth (i.e., improved resistance to abrasion). This behavior can be attributed to the fact that higher compressive strength reflects a denser and more cohesive internal structure, which is better able to resist surface damage, microcrack initiation, and material removal under mechanical actions such as rotating cutting and sandblasting. Consequently, specimens with higher strength consistently exhibit lower rotating cutter and sandblasting depths. However, this relationship is influenced by aggregate characteristics; specifically, increasing aggregate size or aggregate content tends to enhance abrasion resistance by providing a harder and more wear-resistant surface, while at the same time it may reduce compressive strength due to the formation of a larger and relatively weaker ITZ and increased heterogeneity within the matrix. Therefore, compressive strength can be considered a useful but incomplete indicator of abrasion resistance, particularly when mix design variations alter the balance between mortar volume and aggregate content. In addition, temperature plays a significant role in modulating these trends: lower testing temperatures (− 20 °C and − 10 °C) are associated with higher compressive strengths and reduced abraded depths, whereas higher temperature (20 °C) leads to lower strength and greater abrasion. Given the coupled influence of these interacting parameters and the limited size of the dataset, the observed correlations should be interpreted as indicative trends rather than robust predictive relationships. This limitation is acknowledged, and further research incorporating an expanded dataset is recommended to support more reliable statistical modelling.

The results shown in Fig. 6c also show a strong positive correlation between rotating cutter depth and sandblasting depth, indicating that both parameters consistently reflect the material’s abrasion response. Although both test methods evaluate abrasion resistance, they involve different mechanisms: rotating cutting primarily induces abrasion through direct mechanical shearing and plowing of the surface, whereas sandblasting causes material loss through high-velocity particle impact and localized micro-fracturing. Despite these differences, both methods exhibit similar trends because they are governed by the same intrinsic material properties, particularly compressive strength and internal cohesion. Overall, the findings confirm that compressive strength is the primary governing property, with abrasion resistance improving as strength increases, while aggregate characteristics and temperature introduce secondary but important effects.

### Statistical analysis

The ANOVA results presented in Tables 3 and 4 demonstrate the statistical significance of the investigated parameters (mixture compositions and temperatures) on compressive strength and abrasion resistance measured using both the rotating cutter and sandblasting methods. A significance level of 0.05 was adopted in this study; therefore, factors with p-values lower than 0.05 were considered statistically significant.

#### Effect of mixtures compositions

For compressive strength, the incorporation of FA, SF, and MK produced statistically significant effects compared with the control SCC mixture, with p-values of 0.023, 5.72 × 10⁻⁵, and 1.12 × 10⁻⁴, respectively. Among these SCMs, MK exhibited the strongest statistical influence, reflected by the highest sum of squares (907.74) and a very high F-statistic (228.65), followed by SF with an F-statistic of 320.44. These findings are consistent with the experimentally observed substantial increases in compressive strength associated with the high pozzolanic reactivity of MK and SF, which promote dense C-S-H formation and pore refinement. In contrast, the incorporation of SL did not produce a statistically significant effect on compressive strength (*p* = 0.130), despite the moderate improvement observed experimentally. This suggests that the strength enhancement associated with SL remained within the variability range of the test results. The ANOVA analysis also indicated that increasing coarse aggregate size from 10 mm to 20 mm did not significantly affect compressive strength (*p* = 0.199), although a slight reduction in strength was experimentally observed. Conversely, concrete type showed a statistically significant effect (*p* = 0.010), confirming the superior compressive strength of NC relative to SCC. This result supports the interpretation that vibration-induced compaction in NC contributes to a denser internal structure and lower entrapped air content. Similarly, both the C/F aggregate ratio and binder content significantly influenced compressive strength, with p-values of 0.0048 and 0.00012, respectively. The binder content exhibited the most pronounced effect among all investigated parameters, as evidenced by the highest sum of squares (1101.62). This confirms the dominant role of cementitious material content in governing hydration product formation, particle packing, and matrix cohesion.

For the rotating-cutter abrasion test, FA, SF, and MK exhibited statistically significant effects on abrasion resistance, with p-values of 0.031, 0.042, and 0.035, respectively. These findings are consistent with the experimental results, where FA increased abrasion depth, while SF and MK improved abrasion resistance. In contrast, SL showed a statistically insignificant effect (*p* = 0.091), despite the moderate improvement observed experimentally. The ANOVA results also showed that coarse aggregate size and concrete type had no statistically significant effect on abrasion resistance in the rotating-cutter test, with p-values of 0.173 and 0.341, respectively. Although SCC-S20 and NC exhibited lower abrasion depths than the corresponding control mixtures, the observed differences were not statistically significant. Conversely, both the C/F aggregate ratio and binder content significantly affected abrasion resistance, with p-values of 0.0089 and 0.019, respectively. The C/F ratio exhibited the highest F-statistic (22.69), indicating a strong influence on abrasion performance.

For the sandblasting abrasion test, the statistical trends differed from those obtained using the rotating-cutter method. Among the SCMs, only MK exhibited a statistically significant effect (*p* = 0.017), while FA, SL, and SF showed statistically insignificant effects, with p-values of 0.609, 0.239, and 0.083, respectively, despite the experimental reductions in abrasion depth. Aggregate size and concrete type also remained statistically insignificant, with p-values of 0.307 and 0.559, respectively.

Similar to the rotating-cutter results, both the C/F aggregate ratio and binder content significantly influenced abrasion resistance in the sandblasting test, with p-values of 0.0079 and 0.018, respectively. The C/F ratio exhibited the highest F-statistic (24.19), confirming its strong influence on abrasion resistance under erosive wear conditions.

#### Effect of cold temperatures

The ANOVA results confirmed that the influence of temperature on compressive strength and abrasion resistance became progressively more significant as the exposure temperature decreased from 0 °C to − 10 °C and − 20 °C. In general, the statistical analysis closely supported the experimental trends observed for all investigated properties.

For compressive strength, the temperature reduction to 0 °C produced statistically insignificant effects for nearly all mixtures, indicating that the limited improvements observed at this temperature were not sufficiently large relative to the experimental variability. The only exception was SCC-8SF, which exhibited a statistically significant response (*p* = 0.047), suggesting a greater sensitivity of this mixture to mild subzero exposure. At − 10 °C, all mixtures showed statistically significant increases in compressive strength, confirming that the influence of subzero temperature became clearly distinguishable from normal experimental variation. The corresponding F-statistics increased substantially compared with those at 0 °C. At − 20 °C, all mixtures exhibited highly significant effects, with extremely low p-values and markedly higher F-statistics, confirming that severe subzero temperatures had a pronounced influence on strength development regardless of mixture composition.

The ANOVA results for abrasion resistance measured using the rotating-cutter method showed a similar progression in statistical significance with decreasing temperature. At 0 °C, most mixtures remained statistically insignificant, indicating that the initial reductions in abrasion depth were relatively modest. However, SCC-30FA and SCC-8SF already exhibited significant responses, suggesting that these mixtures were more sensitive to early temperature reduction under grinding abrasion conditions. At − 10 °C, the temperature effect became statistically significant for several mixtures, particularly SCC-30FA, SCC-8SF, SCC-20MK, NC, and NC-C250. The highest F-statistics were observed for SCC-30FA and SCC-8SF. At − 20 °C, all mixtures exhibited statistically significant improvements in abrasion resistance.

For abrasion resistance evaluated using the sandblasting method, the ANOVA results showed a comparable trend, although the statistical significance at 0 °C was generally weaker than that observed in the rotating-cutter test. Only SCC-8SF exhibited a statistically significant response at 0 °C, while the remaining mixtures showed insignificant changes. At − 10 °C, most mixtures became statistically significant, particularly SCC-8SF, SCC-20MK, SCC-30FA, SCC-30SL, NC, and NC-2 C/F, indicating that lower temperatures increasingly influenced resistance to erosive abrasion. At − 20 °C, all mixtures exhibited highly significant reductions in abrasion depth, with SCC-20MK, SCC-8SF, NC-2 C/F, and SCC-S20 showing the highest F-statistics.

**Table 3:** ANOVA results for the effects of mixture compositions on compressive strength and abrasion resistance of concrete mixtures.

Property	Factor	Reference mixture	Compared Mixture	DF	Sum of square	Mean square	F statistic	*P*-value	Significance
Compressive strength	FA	SCC	SCC-30FA	1	25.215	25.215	12.7995	0.02322	Significant
	SL		SCC-30SL	1	12.615	12.615	3.6146	0.1301	Insignificant
	SF		SCC-8SF	1	464.64	464.64	320.4414	5.72E-05	Significant
	MK		SCC-20MK	1	907.74	907.74	228.6499	0.000112	Significant
	Aggregate size		SCC-S20	1	5.415	5.415	2.3646	0.1989	Insignificant
	Concrete type		NC	1	52.215	52.215	21.1826	0.01001	Significant
	C/F ratio	NC	NC-2 C/F	1	168.54	168.54	32.0114	0.00481	Significant
	Binder content		NC-C250	1	1101.615	1101.615	220.323	0.00012	Significant
Abrasion resistance (Rotating cutter method)	FA	SCC	SCC-30FA	1	0.008067	0.008067	10.5492	0.03144	Significant
	SL		SCC-30SL	1	0.0054	0.0054	4.918	0.09085	Insignificant
	SF		SCC-8SF	1	0.00735	0.00735	8.6675	0.04222	Significant
	MK		SCC-20MK	1	0.01215	0.01215	9.9103	0.03458	Significant
	Aggregate size		SCC-S20	1	0.00375	0.00375	2.7372	0.1734	Insignificant
	Concrete type		NC	1	0.00135	0.00135	1.1638	0.3414	Insignificant
	C/F ratio	NC	NC-2 C/F	1	0.01815	0.01815	22.6875	0.008885	Significant
	Binder content		NC-C250	1	0.02535	0.02535	14.3871	0.01922	Significant
Abrasion resistance (Sandblasting method)	FA	SCC	SCC-30FA	1	0.06	0.06	0.3068	0.6091	Insignificant
	SL		SCC-30SL	1	0.24	0.24	1.9072	0.2394	Insignificant
	SF		SCC-8SF	1	0.54	0.54	5.2627	0.08348	Insignificant
	MK		SCC-20MK	1	2.16	2.16	15.389	0.01721	Significant
	Aggregate size		SCC-S20	1	0.24	0.24	1.3698	0.3068	Insignificant
	Concrete type		NC	1	0.06	0.06	0.4045	0.5594	Insignificant
	C/F ratio	NC	NC-2 C/F	1	2.535	2.535	24.1922	0.007938	Significant
	Binder content		NC-C250	1	3.84	3.84	14.7882	0.01837	Significant

**Table 4:** ANOVA results for the effects of temperatures on compressive strength and abrasion resistance of concrete mixtures.

Property	Target Temperature	Reference Temperature	Mixture	DF	Sum of Square	Mean Square	F Statistic	*P*-value	Significance
Compressive strength	0 °C	20 °C	SCC	1	6	6	5.5046	0.07884	Insignificant
			SCC-30FA	1	6	6	3.5608	0.1322	Insignificant
			SCC-30SL	1	7.26	7.26	1.5	0.2879	Insignificant
			SCC-8SF	1	9.375	9.375	8.0472	0.04702	Significant
			SCC-20MK	1	8.64	8.64	1.6908	0.2634	Insignificant
			SCC-S20	1	5.415	5.415	1.7136	0.2607	Insignificant
			NC	1	11.76	11.76	2.5372	0.1864	Insignificant
			NC-2 C/F	1	6.615	6.615	1.0492	0.3636	Insignificant
			NC-C250	1	4.335	4.335	0.6908	0.4526	Insignificant
	-10 °C	20 °C	SCC	1	39.015	39.015	13.6416	0.02096	Significant
			SCC-30FA	1	55.815	55.815	19.3802	0.01167	Significant
			SCC-30SL	1	63.375	63.375	16.9452	0.01465	Significant
			SCC-8SF	1	48.1667	48.1667	54.0187	0.001825	Significant
			SCC-20MK	1	48.735	48.735	12.8588	0.02305	Significant
			SCC-S20	1	43.74	43.74	16.4128	0.01546	Significant
			NC	1	116.16	116.16	38.5274	0.003427	Significant
			NC-2 C/F	1	61.44	61.44	8.1055	0.04654	Significant
			NC-C250	1	50.518	50.518	12.4168	0.02437	Significant
	-20 °C	20 °C	SCC	1	241.935	241.935	162.3724	0.000219	Significant
			SCC-30FA	1	230.64	230.64	97.7288	0.000588	Significant
			SCC-30SL	1	230.64	230.64	42.3971	0.002871	Significant
			SCC-8SF	1	220.8267	220.8267	112.2847	0.000449	Significant
			SCC-20MK	1	208.86	208.86	36.3868	0.003807	Significant
			SCC-S20	1	168.54	168.54	56.557	0.001674	Significant
			NC	1	337.5	337.5	94.9367	0.000621	Significant
			NC-2 C/F	1	174.96	174.96	28.8475	0.005803	Significant
			NC-C250	1	118.815	118.815	15.9805	0.01616	Significant
Abrasion resistance (Rotating cutter method)	0 °C	20 °C	SCC	1	0.00135	0.00135	1.5169	0.2856	Insignificant
			SCC-30FA	1	0.006017	0.006017	8.6611	0.04226	Significant
			SCC-30SL	1	0.00135	0.00135	1.08	0.3574	Insignificant
			SCC-8SF	1	0.0054	0.0054	9.1216	0.03916	Significant
			SCC-20MK	1	0.0054	0.0054	6.5854	0.06223	Insignificant
			SCC-S20	1	0.00135	0.00135	1.2736	0.3222	Insignificant
			NC	1	0.00375	0.00375	4.6875	0.09634	Insignificant
			NC-2 C/F	1	0.0006	0.0006	0.813	0.4182	Insignificant
			NC-C250	1	0.0054	0.0054	2.5328	0.1867	Insignificant
	-10 °C	20 °C	SCC	1	0.0096	0.0096	6.2745	0.06642	Insignificant
			SCC-30FA	1	0.02282	0.02282	113.4207	0.00044	Significant
			SCC-30SL	1	0.0054	0.0054	7.3171	0.05381	Insignificant
			SCC-8SF	1	0.0216	0.0216	59.6685	0.001512	Significant
			SCC-20MK	1	0.0216	0.0216	30.5949	0.00522	Significant
			SCC-S20	1	0.0054	0.0054	4.376	0.1046	Insignificant
			NC	1	0.01815	0.01815	15.6466	0.01674	Significant
			NC-2 C/F	1	0.00375	0.00375	7.6844	0.05022	Insignificant
			NC-C250	1	0.0216	0.0216	11.8162	0.02635	Significant
	-20 °C	20 °C	SCC	1	0.0294	0.0294	28.2692	0.006018	Significant
			SCC-30FA	1	0.03527	0.03527	42.0507	0.002915	Significant
			SCC-30SL	1	0.0216	0.0216	19.6721	0.01137	Significant
			SCC-8SF	1	0.06	0.06	122.9508	0.000376	Significant
			SCC-20MK	1	0.06615	0.06615	85.0257	0.000769	Significant
			SCC-S20	1	0.0294	0.0294	30.4979	0.00525	Significant
			NC	1	0.0384	0.0384	45.1765	0.002552	Significant
			NC-2 C/F	1	0.01815	0.01815	40.3333	0.00315	Significant
			NC-C250	1	0.04335	0.04335	21.1463	0.01004	Significant
Abrasion resistance (Sandblasting method)	0 °C	20 °C	SCC	1	0.96	0.96	6.0603	0.06956	Insignificant
			SCC-30FA	1	0.54	0.54	2.6407	0.1795	Insignificant
			SCC-30SL	1	0.375	0.375	3.195	0.1484	Insignificant
			SCC-8SF	1	0.54	0.54	8.2952	0.04501	Significant
			SCC-20MK	1	0.735	0.735	7.593	0.05108	Insignificant
			SCC-S20	1	0.735	0.735	4.8595	0.0922	Insignificant
			NC	1	1.0417	1.0417	6.0701	0.06941	Insignificant
			NC-2 C/F	1	0.24	0.24	2.4793	0.1905	Insignificant
			NC-C250	1	0.24	0.24	0.7292	0.4413	Insignificant
	-10 °C	20 °C	SCC	1	1.6017	1.6017	10.0057	0.03408	Significant
			SCC-30FA	1	2.535	2.535	14.7746	0.0184	Significant
			SCC-30SL	1	1.215	1.215	14.7667	0.01842	Significant
			SCC-8SF	1	2.535	2.535	57.8741	0.001602	Significant
			SCC-20MK	1	2.535	2.535	29.4247	0.0056	Significant
			SCC-S20	1	0.96	0.96	5.7575	0.0744	Insignificant
			NC	1	1.215	1.215	7.7241	0.04986	Significant
			NC-2 C/F	1	0.96	0.96	14.9696	0.01801	Significant
			NC-C250	1	2.16	2.16	7.2862	0.05413	Insignificant
	-20 °C	20 °C	SCC	1	3.84	3.84	27.3582	0.006381	Significant
			SCC-30FA	1	4.335	4.335	19.3656	0.01169	Significant
			SCC-30SL	1	2.535	2.535	20.1446	0.01092	Significant
			SCC-8SF	1	5.415	5.415	68.8493	0.001152	Significant
			SCC-20MK	1	6.615	6.615	85.421	0.000762	Significant
			SCC-S20	1	5.415	5.415	42.9482	0.002803	Significant
			NC	1	4.86	4.86	43.8486	0.002697	Significant
			NC-2 C/F	1	2.94	2.94	53.9945	0.001827	Significant
			NC-C250	1	4.86	4.86	13.7552	0.02068	Significant

## Conclusions

This study investigated the abrasion resistance of concrete for cold-region infrastructure, with a focus on the effects of temperature and mixture composition. Both self-consolidating concrete (SCC) and normal concrete (NC) were tested at four representative temperatures (+ 20 °C, 0 °C, − 10 °C, and − 20 °C) typical of northern terrestrial environments. The mixtures included different supplementary cementing materials—namely metakaolin (MK), silica fume (SF), slag (SL), and fly ash (FA)—along with variations in aggregate sizes, coarse-to-fine (C/F) ratios, and binder contents. Abrasion resistance was evaluated using rotating cutter and sandblasting methods to ensure a thorough assessment of surface durability. The results demonstrate the combined effects of temperature and mixture design on abrasion performance. Based on these results, the following conclusions are drawn:

1. Mixtures incorporating MK and SF consistently demonstrated superior mechanical performance, achieving the highest compressive strength and the greatest resistance to abrasion under all temperature conditions. This behavior is attributed to their high pozzolanic reactivity and ability to refine the pore structure, leading to a denser and more cohesive cementitious matrix. SL showed moderate improvements in performance, while FA exhibited comparatively lower performance, particularly at room temperature. However, all SCM-based mixtures benefited more noticeably from subzero temperatures, which enhanced their overall mechanical response.
2. Increasing the maximum coarse aggregate size slightly reduced compressive strength at 20 °C, while it improved abrasion resistance, reflecting the greater contribution of larger, hard aggregate particles to surface wear resistance. At subzero temperatures, both compressive strength and abrasion resistance further improved, indicating that lower temperatures enhance overall mechanical performance regardless of aggregate size.
3. Although NC and SCC had the same composition, NC consistently showed higher compressive strength and abrasion resistance across all temperatures. This is attributed to the more effective densification achieved through mechanical vibration, which produces a tighter aggregate packing and a more cohesive internal structure. In contrast, SCC tends to form a more mortar-rich surface due to its high flowability, making it comparatively more vulnerable to abrasion. Under subzero conditions, both concretes showed enhanced performance, with NC benefiting more strongly from cold-induced densification effects.
4. A higher C/F aggregate ratio led to a trade-off between compressive strength and abrasion resistance. The reduction in compressive strength is associated with increased ITZ extent and reduced matrix cohesion, while abrasion resistance improved due to the greater contribution of hard coarse aggregates at the surface. Under subzero temperatures, both compressive strength and abrasion resistance improved, confirming that cold conditions enhance overall mechanical performance even in mixtures with higher coarse aggregate content.
5. Reducing binder content had the most detrimental effect on overall concrete performance, leading to lower strength and poorer abrasion resistance due to reduced paste volume and weaker matrix cohesion. Although subzero temperatures improved both compressive strength and abrasion resistance, the low-binder mixture remained the least durable across all conditions, indicating that temperature effects cannot offset insufficient cementitious content.
6. From a practical perspective, mixture selection for cold-region concrete infrastructure should be based on the dominant performance requirement. For applications exposed to severe abrasion, such as pavements and bridge decks subjected to studded tires or sand erosion, mixtures containing SF or MK with higher coarse aggregate content are recommended due to their superior abrasion resistance at subzero temperatures. Although higher coarse aggregate content may slightly reduce compressive strength, it significantly enhances resistance to mechanical and erosive wear. Conversely, for applications governed primarily by compressive strength, mixtures incorporating MK or SF with balanced aggregate proportions provide the best overall performance under cold-temperature exposure.
7. The ANOVA results confirm that concrete performance is significantly governed by both mixture composition and temperature, with the level of statistical influence varying depending on the property and loading mechanism. Among mixture parameters, SCM type (particularly MK and SF), C/F aggregate ratio, and binder content were the most influential factors, showing consistent statistical significance in both compressive strength and abrasion resistance, while aggregate size and concrete type had comparatively limited or inconsistent effects. In addition, the influence of temperature was strongly dependent on the severity of exposure, being generally insignificant at 0 °C but becoming increasingly significant at − 10 °C and reaching a consistently dominant and highly significant effect at − 20 °C across all mixtures and test methods.

Overall, subzero temperatures generally enhance both compressive strength and abrasion resistance for all mixtures, with the magnitude of improvement strongly influenced by microstructural density and binder content. Low-binder and FA-rich mixtures exhibited the highest proportional strength gains at − 20 °C, yet their absolute performance remained the lowest, indicating that ice formation cannot fully compensate for low cement content or high porosity. Aggregate characteristics also played a key role: increasing the maximum coarse aggregate size slightly reduced compressive strength but improved abrasion resistance, whereas higher C/F aggregate ratios had a stronger influence, either decreasing strength or enhancing abrasion. However, both aggregate size and content showed comparable sensitivity to subzero temperatures, with their effects on strength and abrasion amplified under cold conditions.

## Limitations of the study and future recommendations

This study has several limitations that should be acknowledged. First, the interpretations related to pore refinement, matrix densification, and ice-induced strengthening were based primarily on mechanical performance observations and literature findings, without direct microstructural characterization such as scanning electron microscopy (SEM), X-ray diffraction (XRD), mercury intrusion porosimetry (MIP), or computed tomography (CT) scanning. Second, the investigation was limited to short-term laboratory testing under low-temperature static conditions and did not consider long-term field exposure, repeated freeze–thaw cycling, moisture variations, deicing salts, or environmental deterioration. Therefore, future research should evaluate the long-term durability of these mixtures under combined freeze–thaw and abrasion conditions representative of real cold-region infrastructure. In addition, the discussed mechanisms associated with ice formation and cold-induced enhancement of internal cohesion were not directly validated experimentally, and the observed improvements may change after repeated freeze–thaw cycles due to progressive microcracking and internal damage. Future studies incorporating cyclic freezing and thawing and/or salt scaling together with microstructural characterization techniques are recommended to better understand the evolution of pore structure and damage mechanisms over time. Finally, the study considered only a limited range of coarse aggregate sizes, C/F ratios, and binder contents; therefore, the findings may not fully represent the behavior of all concrete mixture configurations used in cold-region infrastructure.

## Data Availability

All data generated or analyzed during this study are included in this published article.
